# Reduced Expression of *PLCXD3* Associates With Disruption of Glucose Sensing and Insulin Signaling in Pancreatic β-Cells

**DOI:** 10.3389/fendo.2019.00735

**Published:** 2019-11-06

**Authors:** Hayat Aljaibeji, Debasmita Mukhopadhyay, Abdul Khader Mohammed, Sarah Dhaiban, Mahmood Y. Hachim, Noha M. Elemam, Nabil Sulaiman, Albert Salehi, Jalal Taneera

**Affiliations:** ^1^Sharjah Institute for Medical Research, University of Sharjah, Sharjah, United Arab Emirates; ^2^Department of Clinical Sciences, Lund University Diabetes Centre (LUDC), Lund University, Malmö, Sweden

**Keywords:** type 2 diabetes, microarray, gene expression, RNA sequencing, PLCXD3

## Abstract

Previous work has shown that reduced expression of *PLCXD3*, a member of the phosphoinositide-specific phospholipases (PI-PLC) family, impaired insulin secretion with an unclear mechanism. In the current study, we aim to investigate the mechanism underlying this effect using human islets and rat INS-1 (832/13) cells. Microarray and RNA sequencing data showed that *PLCXD3* is among the highly expressed PI-PLCs in human islets and INS-1 (832/13) cells. Expression of *PLCXD3* was reduced in human diabetic islets, correlated positively with *Insulin* and *GLP1R* expression and inversely with the donor's body mass index (BMI) and glycated hemoglobin (HbA_1c_). Expression silencing of *PLCXD3* in INS-1 (832/13) cells was found to reduce glucose-stimulated insulin secretion (GSIS) and insulin content. In addition, the expression of *Insulin, NEUROD1, GLUT2, GCK, INSR, IRS2*, and *AKT* was downregulated. Cell viability and apoptosis rate were unaffected. In conclusion, our data suggest that low expression of *PLCXD3* in pancreatic β-cells associates with downregulation of the key insulin signaling and insulin biosynthesis genes as well as reduction in glucose sensing.

## Introduction

Phosphoinositide-specific phospholipase C (PI-PLC) is an enzyme that hydrolyzes the membrane phospholipid phosphatidylinositol-4,5-bisphosphate (PIP_2_) to inositol-1,4,5-trisphosphate (IP_3_) and diacylglycerol in response to external stimuli such as hormones, neurotransmitter, and growth factors (1). There are six different subtypes of PI-PLCs (β, γ, δ, ε, ζ, and η) that have been identified and isolated from different tissues in humans, including brain, heart, spleen, thymus, and hematopoietic cells (2). The majority of the PI-PLC subtypes (with an exception of *PLC-*β and *PLC-*γ) have a C2 domain (calcium-binding motif), which plays an essential role in membrane interactions (2). Additionally, all PI-PLC subtypes hold distinct tissue distribution patterns with a distinctive function in regulating cell metabolism (3). In human islets, little is known about the expression of PI-PLC subtypes. However, it has been previously reported that rodent pancreatic islets contain the three major PLC subtypes classes (β1, γ1, and δ1). Various studies have linked the PI-PLC with the regulation of insulin secretion and pancreatic β-cell function, where calcium influx was shown to activate PI-PLC in a positive feedback manner resulting in exocytosis (4–6).

In 2012, Gellatly et al. have identified a new class of PI-PLC, which contains only a catalytic X domain in its structure termed phospholipase C X domain containing protein (*PLCXD3*) (3). Three different isoforms of *PLCXD* (*PLCXD1, PLCXD2*, and *PLCXD3*) have been recognized; each has a distinct function depending on their tissue distribution and cellular localization. Furthermore, it was found that *PLCXD* proteins function as active phosphodiesterases as shown by the increase of inositol phosphate turnover (3). The *PLCXD3* isoform was shown to localize in cytoplasmic and perinuclear vesicles in HeLa cells. At the messenger RNA (mRNA) level, *PLCXD3* expression was found to be predominant in the brain (3). Additionally, *PLCXD3* has been ascribed a role in early-onset bipolar disorder vulnerability, sporadic Creutzfeldt–Jakob disease and the mouse olfactory sensory neurons (7–9). Moreover, a mutation in the *PLCXD3* gene has been linked to rapid-onset obesity with hypothalamic dysfunction, hypoventilation, and autonomy dysregulation (ROHHAD) (10).

In previous work, siRNA silencing of *PLCXD3* in INS-1 (832/13) cells markedly reduced in glucose-stimulated insulin secretion (GSIS), indicating that the gene is a key molecule in the pancreatic β-cell function (11). However, how *PLCXD3* mediates insulin secretion impairment is not fully understood. In the present study, we shed more light on the expression profile of the PI-PLC subtypes overall and *PLCXD3* in particular using microarray and RNA sequencing expression data from isolated human islets and INS-1 (832/13) cells. Further, we performed several functional studies to investigate the consequences of *PLCXD3* silencing on insulin biosynthesis, insulin secretion, cell viability, apoptosis, and expression of key β-cell function genes.

## Materials and Methods

### Human Pancreatic Islets

Islets were obtained from the Human Tissue Laboratory at Lund University Diabetes Centre in collaboration with the Nordic Network for Clinical Islet Transplantation (Uppsala University, Sweden) (12). Islets were collected from 170 donors with no history of diabetes (65 females and 105 males, age 58.9 ± 10, BMI 26.2 ± 3.5, HbA_1c_ 5.7 ± 0.7, days in culture 3.5 ± 1.9) and 32 donors with type 2 diabetes (T2D) (10 females and 22 males, age 61.7 ± 11, BMI 28.1 ± 4.5, HbA_1c_ 7.0 ± 1.2, and days in culture 2 ± 1.0). Written informed consent has been obtained from the patients or relative in accordance with the Declaration of Helsinki. The local ethics committees at both Uppsala and Lund universities (Sweden) have approved all procedures with human islets isolation and investigations (approval number: 2011–5).

### Microarray Gene Expression

The Affymetrix gene expression array (Human Gene 1.0 ST, *n* of donors = 67 non-diabetics + 10 with T2D; and Rat 2.0 ST, *n* = 3) was performed as previously described (12). The robust multi-array analysis (RMA) method was used to normalize array data. The background values of the arrays were set based on the mean expression values of all negative probe sets. Genes with expression levels higher than the background values are likely to be expressed, whereas genes with expression levels lower than the background values are not. Human expression data are deposited in a Gene Expression Omnibus (GEO) database with accession numbers GSE 50398 and GSE 50397.

### RNA Sequencing Expression of *PLCXD3* in Isolated Human Islets

RNA sequencing was performed (non-diabetic = 170 and T2D = 32) using Illumina's TruSeq as described previously (13). The human reference genome (hg19) was used to align the output reads with STAR (14). Expression data were presented as fragments/kilobase of exon per million fragments mapped (FPKM) or transformed into log2 counts per million using the voom-function (edgeR/limma R packages).

#### RNA Sequencing in INS-1 (832/13) Cells

The RNA sequencing was performed on a BGISEQ-500 RS platform at the Beijing Genomics Institution (BGI, Shenzhen, China) (www.genomics.org.cn). Briefly, RNAs (from control and treated cells, three replicates for each treatment) were fragmented to prepare complementary DNA (cDNA) libraries using the RNA fragments as templates for N6 random primers. The quantity and quality of the cDNA libraries were assessed using an Agilent 2100 Bioanalyzer. Finally, the libraries were sequenced on the BGISEQ-500 with 50 single-end reads. Sequencing reads that contained adapters, had low quality, or aligned to rRNA were filtered off before mapping. Clean reads were aligned to the hg19 UCSC RefSeq (RNA sequences, GRCh37) using bowtie2. Fragments per kilobase of transcript per million mapped reads values were obtained by transforming mapped transcript reads using RSEM. Differential expression analysis was performed by DESeq2. Differentially expressed genes were defined as genes with fold change ≥0.8 and *p*-value ≤ 0.05. Clean reads were mapped to the hg19 genome using hisat2.

### siRNA Silencing of *PLCXD3* in INS-1 (832/13) Cells and Insulin Secretion Measurements

The rat clonal pancreatic INS-1 (832/13) β-cells (a kind gift from Dr. Chris Newgard, Duke University) were maintained in RPMI-1640 medium (15). INS-1 (832/13) cells were cultured in a 24-well plate in complete RPMI-1640 medium without antibiotics overnight and then were transfected with two different siRNA sequences for *PLCXD3* (s160317 and s160319) (Thermo Fisher Scientific, USA) along with the Lipofectamine 3000 transfection reagent and siRNA negative control sequence at a concentration of 40 nM (Thermo Fisher Scientific) (16). After 48 h, cells were washed with pre-warmed secretion assay buffer (11, 17) containing 2.8 mM glucose and then pre-incubated with the same buffer for 2 h. Next, the buffer was removed, and cells were incubated with 1 ml of SAB containing either 2.8 or 16.7 mM glucose for 1 h. Stimulated insulin secretion was assessed using a rat insulin ELISA kit (Elabsciences, China). For insulin content measurements, the total protein was extracted from transfected cells using an M-PER reagent (Thermo Fisher Scientific, USA) and quantified by Pierce BCA protein assay kit (Thermo Fisher Scientific, USA). The total protein was diluted (1:250), and insulin content was assessed using ELISA (Elabsciences). Finally, insulin content was normalized to the total amount of protein.

To evaluate the effect of hyperglycemia exposure on the expression of *PLCXD3*, INS-1 (832/13) cells were cultured for 24 h at 11.1 mM (control) and 16.7 and 22.2 mM glucose at mRNA and protein levels.

### Quantitative RT-qPCR

Extraction of total RNA from intact human pancreatic islets was performed using the RNeasy Plus Mini Kit (Qiagen, Germany). RNA quantity and quality were measured by NanoDrop 2000 and Agilent 2100 Bioanalyzer. For cDNA synthesis, a high-capacity cDNA synthesis kit was used (Thermo Fisher Scientific, USA). Silencing efficiency and gene expression analysis were assessed using TaqMan gene expression assays; *PLCXD3* (Rn01762608_m1), *INS1* (Rn02121433_g1), *INS2* (Rn01774648_g1), *MAFA* (Rn00845206_g1), *PDX1* (Rn00755591_m1), *INSR* (Rn00690703_m1), *GCK* (Rn00561265_m1), *GLUT2* (Rn00563565_m1). *HPRT1* (Rn01527840_m1) was used as an endogenous control to normalize the expression of target mRNA. SYBR Green qPCR gene expression analysis with the corresponding primers (Table 1) was used for *NEUROD1, NKX2.2*, and *HPRT1*. Relative gene expression was performed using the 2^−ΔΔ*Ct*^ method. All reactions were executed in QuantStudio 3 Real-time PCR (Applied Biosystems, USA).

**Table 1:** SYBR Green qPCR primer sequences.

**Primer**	**Gene bank ID**	**Forward primer**	**Reverse primer**	**Size**
HPRT1	NM_012583.2	TTGTGTCATCAGCGAAAGTGG	CACAGGACTAGAACGTCTGCT	120 bp
NEUROD1	NM_019218.2	CCCTAACTGATTGCACCAGC	TGCAGGGTAGTGCATGGTAA	137 bp
NKX2.2	NM_001191904.1	CGAATTGACCAAGTGAGGCT	TTCTTCATCGTTGGTGCCG	130 bp

### Apoptosis and MTT Assays

The impact of *PLCXD3* on INS-1 (832/13) cell apoptosis rate was evaluated using annexin-V staining in *PLCXD3*-silenced cells compared to control cells using flow cytometry (FACSAria III, BD Biosciences, USA) as described previously (18). Cell viability was assessed using MTT colorimetric assay (Sigma-Aldrich, Germany) in transfected cells as described previously (18).

### Western Blot Analysis

Total protein was extracted using an M-PER reagent containing protease inhibitor cocktail (Thermo Fisher Scientific, USA). A lysate containing a 30–40 μg sample of total protein was separated by SDS-PAGE (10%) and transferred onto a nitrocellulose membrane (Bio-Rad, USA). The membranes were blocked with 5% skimmed milk prepared in Tris-buffered saline with 0.1% Tween 20 (TBS-T) for 1 h. The blot was probed with *PLCXD3* (anti-rabbit; #PA5-71235, Thermo Fisher Scientific), *Insulin* (anti-mouse; #8138s, Cell Signaling Technology, USA), *INSR*α (anti-rabbit; #ab5500, Abcam), *PDX1* (anti-rabbit; #ab47267, Abcam), *GLUT2* (anti-rabbit; #ab54460, Abcam), AKT1/2/3 (anti-Rabbit; #Ab179463, Abcam), IRS2 [Phospo (s731), #ab3690, Abcam], NeuroD1 (anti-rabbit, #ab213725, Abcam), or *GCK* (anti-rabbit; #ab37796, Abcam) antibody overnight at 4°C with the same procedure probing with primary antibodies against β-actin (Sigma-Aldrich). The secondary horseradish peroxidase (HRP)-linked anti-mouse (#7076S, Cell Signaling Technology, USA) and HRP-linked anti-rabbit (#7074S, Cell Signaling) antibodies were added to the membranes at 1:1,000 dilutions for 1 h. Chemiluminescence was detected using the Clarity ECL substrate kit (Bio-Rad, USA). Protein bands were quantified using the Bio-Rad Image Lab software (ChemiDoc™ Touch Gel Western Blot Imaging System; Bio-Rad, USA). β-Actin was used as an endogenous control.

### Statistical Analysis

The edge-R was used to calculate differential gene expression (adjusted for age, sex, and BMI) in human islets. Correlation of gene expression and phenotypes was calculated by a linear regression model adjusted for sex, age, and BMI or Spearman's test. *p*-values illustrating the significance were calculated using the eBayes function in limma (19). For insulin secretion and qRT-PCR analysis, we used a parametric unpaired two-tailed Student's *t*-test. Data are presented as mean ± SEM unless otherwise stated. Statistical significance was indicated by asterisks (^*^p < 0.05, ^**^p < 0.01, ^***^p < 0.001).

## Results

### Microarray Expression Profile of PI-PLC Subtypes in Human and Rat Cells

**Figure 1:**
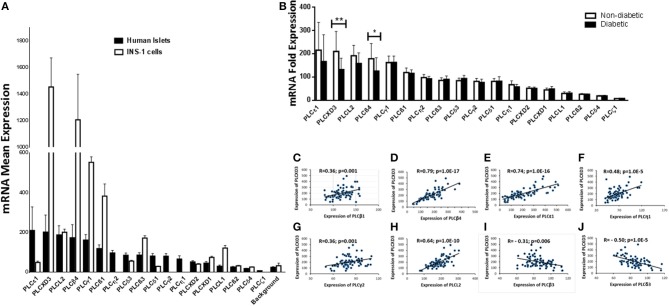
Microarray gene expression profile of phosphatidylinositol-specific phospholipase C (PI-PLC) subtypes in non-diabetic human islets and INS-1 (832/13) cells. **(A)** Mean expression of 18 human (*n* = 67) and 14 rat (*n* = 3) PI-PLC subtypes that present in the microarray. The background is the mean expression of all negative control probe sets on the human or rat whole transcript arrays. **(B)** Differential expression analysis of the 18 human PI-PLC subtypes in diabetic islets (*n* = 10) vs. non-diabetic islets (*n* = 67) donors. **(C–J)** Co-expression correlations of *PLCXD3* (*n* = 77) with *PLC*β*1* **(C)**, *PLC*β*4* **(D)**, *PLC*ε*1* **(E)**, *PLC*η*1* **(F)**, *PLC*γ*2* **(G)**, *PLCL2* **(H)**, *PLC*β*3* **(I)**, and *PLC*δ*3* **(J)**. *R* and *p* values are indicated in the respective graphs. *R*: correlation coefficient; *p*: *p*-value. *P < 0.05, **P < 0.01, and ***P < 0.001. Bars represent mean ± SD.

Expression of PI-PLC subtypes in human pancreatic islets is not well-characterized. Thus, using microarray expression from non-diabetic islets (*n* = 67), we examined the expression of 18 PI-PLC subtypes. *PLC*ε*1* and *PLCXD3* were the most highly expressed (Figure 1A). However, *PLC*δ*4* and *PLC*ζ*1* had low expression as their expression levels were similar to/below the background level (Figure 1A). Microarray expression from rat INS-1 (832/13) cells showed that 14 transcripts of PI-PLC subtypes were present. *PLC*η*1, PLC*η*2, PLC*γ*2*, and PLCζ1 transcripts were not present in the Rat Gene 2.0 ST array (Figure 1A). Among the PI-PLC subtypes, *PLCXD3* and *PLC*β*4* were the highest expressed genes in INS-1 (832/13) cells, whereas *PLC*δ*4, PLC*β*2*, and *PLC*δ*4* were the lowest (Figure 1A). Interestingly, although *PLC*ε*1* was highly expressed in human islets, its expression was low in INS-1 (832/13) cells (Figure 1A). Next, differential expression analysis for PI-PLC subtypes revealed a significantly lower expression for *PLCXD3* (*p* = 0.002) and *PLC*β*4* (*p* = 0.02) in diabetic islets when compared with non-diabetic ones (Figure 1B). As the focus of this study is *PLCXD3*, we analyzed the co-expression correlation of *PLCXD3* with all PI-PLC subtypes in human islets. Our data showed that *PLCXD3* is correlated positively with *PLC*β*1, PLC*β*4, PLC*ε*1, PLC*η*1, PLCL2*, and *PLC*γ*2* and negatively with *PLC*β*3* and *PLC*δ*3* (Figures 1C–J).

### RNA Sequencing Expression Analysis of *PLCXD3* From Isolated Human Islets

**Figure 2:**
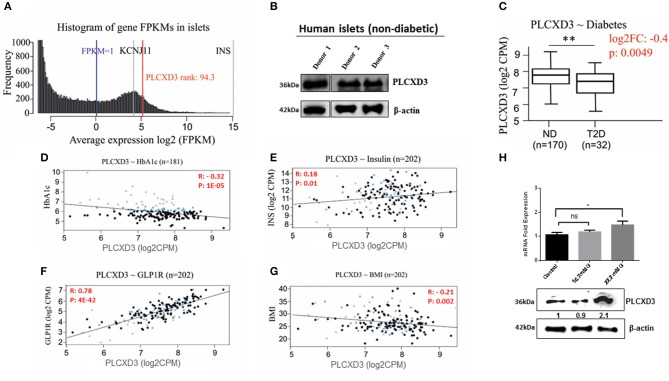
RNA sequencing expression of *PLCXD3* in human islets. **(A)** Expression histogram of *PLCXD3* obtained from non-diabetic human islets (*n* = 170) compared to *KCNJ11* and *INS* genes. **(B)** Western blot expression analysis of *PLCXD3* in human islets obtained from three different non-diabetic donors. **(C)** Expression level of *PLCXD3* in diabetic islets (*n* = 32) vs. non-diabetic islets (*n* = 170). Correlation of RNA sequencing expression of *PLCXD3* with **(D)** HbA_1c_ levels, **(E)** insulin transcript, **(F)** *GLP1R* transcript, **(G)** and body mass index (BMI). **(H)** messenger RNA (mRNA) and protein expression analysis of *PLCXD3* measured from cultured INS-1 (832/13) cells for 24 h at 16.7 mM glucose. The correlation coefficient (*R*) and *p*-value are indicated in the respective graphs (*n* = 192). *P < 0.05, **P < 0.01, and ***P < 0.001. Bars represent mean ± SEM.

To further validate the *PLCXD3* expression in human islets, RNA sequencing data from a large number of donors (*n* = 202) were analyzed. As illustrated in Figure 2A, *PLCXD3* showed high expression (top 94th percentile) as compared with the expression of *KCNJ11*, a functional marker of human islets (20). Furthermore, expression of *PLCXD3* was confirmed in non-diabetic human islets (*n* = 3) at the protein level (Figure 2B).

In a previous study, we reported lower expression of *PLCXD3* in diabetic islets compared to healthy islets, where its expression was correlated with insulin secretion and HbA_1c_ (11). This finding was further confirmed using RNA sequencing data collected from a large number of donors (*n* = 202). As shown in Figure 2C, significant reduction of *PLCXD3* expression in diabetic islets (*p* = 0.004) compared to non-diabetic islets was observed. Furthermore, *PLCXD3* expression was shown to correlate positively with insulin expression (*p* = 0.01) and negatively with HbA_1c_ (*p* = 0.0001) (Figures 2D,E). Additionally, *PLCXD3* exhibited a strong positive correlation with *GLP1R* (*p* = 4E−42) (Figure 2F) and a negative correlation with BMI (*p* = 0.002) (Figure 2G). Finally, to investigate whether the reduced expression of *PLCXD3* was due to the exposure of hyperglycemia status, expression of *PLCXD3* was measured in INS-1 (832/13) cells cultured for a short term (24 h) at 11.1 mM (control) and 16.7 or 22.2 mM glucose at mRNA and protein levels. As illustrated in Figure 2H, the *PLCXD3* expression level was significantly unchanged at 16.7 mM glucose as compared to control cells, while at 22.2 mM glucose, we observed increased *PLCXD3* expression (Figure 2H), indicating that the observed reduction of *PLCXD3* expression in diabetic islets is not due to short-term hyperglycemia.

### *PLCXD3* Silencing in INS-1 (832/13) Cells Impairs Insulin Secretion and Content

**Figure 3:**
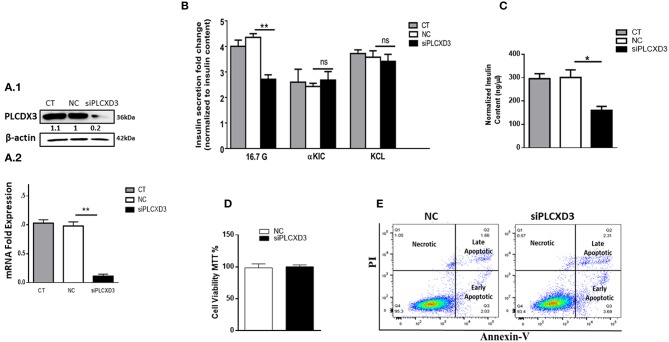
Silencing of *PLCXD3* in INS-1 (832/13) cells reduces insulin secretion and content. **(A.1)** Silencing efficiency of small interfering RNA (siRNA) of *PLCXD3* 48 h after transfection with a pool of two different siRNA sequences measured by RT-PCR. **(A.2)** Fold change differences in the intensity of the western blot band of *PLCXD3* protein expression relative to the endogenous control β-actin in *PLCXD3*-silenced cells compared to untreated control (CT) or negative siRNA control (NC) 48 h post-transfection. **(B)** Stimulated insulin secretion in response to 2.8 mM glucose, 16.7 mM glucose, and 10 mM α-KIC in the presence of 2.8 mM glucose or 35 mM potassium chloride (KCl) in the presence of 2.8 mM glucose performed in *PLCXD3*-silenced cells or negative control cells for one static hour of incubation. Insulin secretion measurements were normalized to protein content. **(C)** Normalized insulin content measurements from *PLCXD3-*silenced cells compared to negative control cells. **(D)** Percentage of cell viability in *PLCXD3*-silenced cells as determined using the MTT assay compared to control cells. **(E)** Assessment of apoptosis in *PLCXD3*-silenced cells and negative control as analyzed by flow cytometry. Data are shown from three independent experiments. *P < 0.05, **P < 0.01, and ***P < 0.001. Bars represent mean ± SD.

In an attempt to explore the mechanistic defect of *PLCXD3* on insulin secretion, we silenced the expression of *PLCXD3* using a pool of two different siRNAs. Silencing efficiency assessed 48 h post-transfection by qRT-PCR showed that almost 85% decreased in *PLCXD3* expression (*p* < 0.01) relative to the negative control (Figure 3A). At the protein level, a comparable expression reduction of *PLCXD3* (~75%; *p* < 0.01) was observed as assessed by western blot analysis (Figure 3A).

Intriguingly, siRNA silencing of *PLCXD3* resulted in a marked reduction in GSIS at 2.8 mM (basal) or 16.7 mM glucose (stimulation) for 1 h (*p* = 0.02, *p* = 0.007, respectively) (Figure 3B) as compared with negative siRNA control.

Moreover, stimulating *PLCXD3*-silenced cells with either 10 mM α-KIC (a secretagogue that directly stimulates mitochondrial metabolism and enhances the mitochondrial ATP synthesis) or 35 mM KCl (depolarizing agent) for 1 h showed no effect on insulin secretion (Figure 3B). However, insulin content was significantly reduced in the transfected cells (~45%; *p* < 0.01) compared to the negative control cells (Figure 3C). Whether *PLCXD3* silencing is associated with decreased cell viability and thereby led to the impairment of GSIS was assessed. As shown in Figure 3D, the percentage of viable cells in *PLCXD3*-silenced cells was not affected compared to control cells. This result was further confirmed by apoptosis analysis with “annexin-V and PI” assay. No difference in the percentage of apoptotic cells was observed in *PLCXD3*-silenced cells as compared with negative control cells (Figure 3E).

Finally, to address whether the impairment of insulin secretion is mainly attributed to reduced expression of *PLCXD3* or due to other subtypes (in particular *PLC*ε*1, PLC*β*4*, and *PLC*γ*1*) which are significantly present in human islets or INS-1 (832/13) cells, we analyzed the expression of all PI-PLC subtypes using RNA sequencing data from *PLCXD3*-silenced INS-1 cells. As shown in Table 2, only *PLC*β*1* was shown to be affected by silencing of *PLCXD3* (FC = 0.66; *p* adj = 0.05).

**Table 2:** RNA sequencing expression of PI-PLC subtypes in *PLCXD3*-silenced cells.

**Symbol**	**Ctrol-exp**	**PLCXD3_Exp**	**log2 fold change**	***p* value**	***p* adj**	**Description**
**Plcxd3**	330.55	101.61	−1.702	0.002	0.050	Phospholipase C, X domain containing 3
**Plcd1**	3.76	2.85	–0.400	0.463	0.949	Phospholipase C, delta 1
**Plcxd2**	24.70	20.79	–0.249	0.547	0.949	Phospholipase C, X domain containing 2
**Plcb2**	0.90	0.78	–0.203	0.555	0.949	Phospholipase C, beta 2
**Plcl2**	805.15	701.67	–0.198	0.423	0.949	Phospholipase C-like 2
**Plcd3**	3.95	3.64	–0.118	0.832	0.984	Phospholipase C, delta 3
**Plch2**	2.01	1.95	–0.045	0.933	0.999	Phospholipase C, eta 2
**Plcb4**	1145.03	1148.07	0.004	0.984	0.999	Phospholipase C, beta 4
**Plcd4**	16.76	16.92	0.014	0.975	0.999	Phospholipase C, delta 4
**Plch1**	104.20	108.63	0.060	0.849	0.986	Phospholipase C, eta 1
**Plcb3**	518.80	552.14	0.090	0.684	0.954	Phospholipase C beta 3
**Plce1**	26.67	29.16	0.129	0.731	0.970	Phospholipase C, epsilon 1
**Plcg2**	4.99	6.16	0.306	0.582	0.949	Phospholipase C, gamma 2
**Plcxd1**	46.44	59.20	0.350	0.256	0.904	Phospholipase C, X domain containing 1
**Plcg1**	1241.31	1626.78	0.390	0.009	0.217	Phospholipase C, gamma 1
**Plcb1**	534.53	844.49	0.660	0.001	0.050	Phospholipase C beta 1
**Plcl1**	548.83	967.15	0.817	0.004	0.140	Phospholipase C-like 1

### *PLCXD3* Signaling in INS-1 (832/13) Cells Affects β-Cell Function Genes

**Figure 4:**
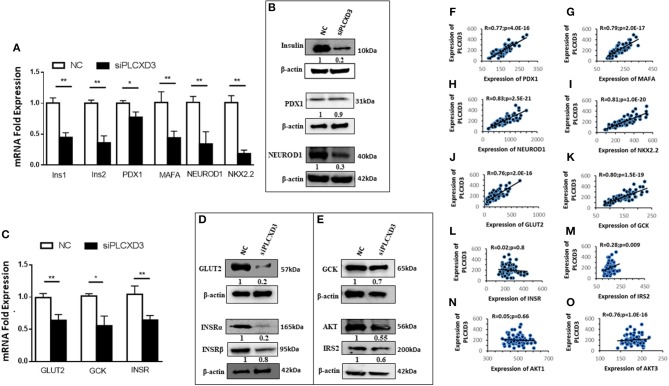
Effect of *PLCXD3* silencing on genes involved in the β-cell function. RNA and protein were extracted from *PLCXD3*-silenced cells or negative control 48 h post-transfection. **(A)** qRT-PCR expression analysis of *Ins1, Ins2, PDX1, MAFA, NEUROD1*, and *NKX2.2*. **(B)** Western blot analysis of *Insulin, PDX1*, and *NEUROD1* relative to the endogenous control protein β-actin. **(C)** qRT-PCR expression analysis of *GLUT2, GCK*, and *INSR*. **(D)** Western blot analysis of *GLUT2* and *GCK* relative to the endogenous control. **(E)** Western blot analysis of *INSR*α, phosphor-*IRS2*, and *AKT1*/*2*/*3* relative to the endogenous control. qRT-PCR data were obtained from three independent experiments, and western blot data are a representative from three independent experiments. **(F–O)** Spearman's expression correlation of *PLCXD3* with *PDX1* **(F)**, *MAFA* **(G)**, *NEUROD1* **(H)**, *NKX2.2* **(I)**, *GLUT2* **(J)**, *GCK* **(K)**, *INSR* **(L)**, *IRS2* **(M)**, *AKT1* **(N)**, and *AKT3* **(O)** using microarray data from human pancreatic islets (*n* = 64). *R* and *p* values are indicated in the respective graphs. *R*, correlation coefficient; *p, p*-value. *P < 0.05, **P < 0.01, and ***P < 0.001. Bars represent mean ± SD.

To further understand how silencing of *PLCXD3* impairs insulin secretion, we analyzed the expression of several genes involved in the β-cell function. At first, genes involved in proinsulin biosynthesis [*Ins1* (*p* = 0.02), *Ins2* (*p* = 0.004), *PDX1* (*p* = 0.03), *MAFA* (*p* = 0.004), *NEUROD1* (*p* = 0.001), and *NKX2.2* (*p* = 0.001)] was shown to be significantly downregulated at the transcriptional level (Figure 4A). At the protein level, a marked expression reduction (*p* < 0.05) of *insulin* (~75%) and *NEUROD1* (~70%) was observed (Figure 4B), whereas *PDX1* expression was not affected (Figure 4B). Glucose-sensing genes showed a significant downregulation [*GLUT2* (*p* = 0.003) and *GCK* (*p* = 0.02)] at the mRNA level (Figure 4C). Similarly, protein expression analysis revealed a marked reduction of *GLUT2* (~75%) and *GCK* (~30%) (*p* < 0.05) (Figure 4D). Genes involved in insulin signaling including INSR (α/β), AKT, and IRS2 revealed that *INSR* mRNA expression is reduced (*p* < 0.05) in *PLCXD3*-silenced cells compared to negative control cells (Figure 4B). At the protein level, a reduced expression of INSRα (~80%, *p* < 0.05), INSRβ (~30%, *p* < 0.05), IRS2 phosphorylation (~40%, *p* < 0.05), and AKT1/2/3 (~40%, *p* < 0.05) was observed in *PLCXD3*-silenced cells (Figure 4E). Moreover, expression correlation analyses showed that *PLCXD3* is positively correlated with expression of *PDX1, MAFA, NEUROD1, NKX2.2, GLUT2, GCK, IRS2*, and *AKT3* (Figures 4F–O) as analyzed in human islet microarray expression data. No correlation was observed between *PLCXD3* and *INSR* or *AKT1* (Figures 4I–N).

## Discussion

Multiple studies have attempted to identify and understand the role of PI-PLC subtypes in human physiology. However, only a few studies have focused on the pathophysiology role of PI-PLCs in diabetes or their respective expression in human pancreatic islets. Thus, in this study, the expression of 18 PI-PLC subtypes in human islets (healthy or diabetic condition) and in the rat INS-1(832/13) cells was examined using microarray analysis. Our data showed that *PLC*ε*1, PLCXD3, PLCL2*, and *PLC*β*4* were the most expressed PI-PLC subtypes in human islets while *PLCXD3* and *PLC*β*4* were the most abundant in INS-1 (832/13) cells. The discrepancy in the expression of the PI-PLCs between human and rat islet cells might be ascribed to a species-specific expression. Another possible explanation could be that there is a discrete functional impact of PI-PLCs in immortalized β-cell lines as compared to primary β-cells. Also, such variations could be of great interest and importance for further functional validation. For example, genes that are highly expressed in INS-1 (832/13) cells such as *PLCXD3* or *PLC*β*4* would be a suitable target to be investigated in the INS-1 (832/13) research model, unlike the low-expression PLC genes such as *PLC*δ*4* or *PLC*β*2*.

The low expression of *PLCXD3* in human diabetic islets and its correlation with eight PI-PLC subtypes (Figures 1, 2C) raise the question of whether ablation of *PLCXD3* might lead to altering the expression of other PI-PLC subtypes. As shown in Figure 1, *PLC*ε*1* and *PLC*β*4* were found to be highly expressed in human islets, and their expression correlated positively with *PLCXD3*. In contrast to human islets, *PLC*ε*1* was previously reported to be present at low levels in most tissues as compared to other PI-PLC subtypes (21–23). It has been shown that knockout of *PLC*ε*1* in mouse islet led to a disruption in GSIS (21, 24, 25). On the other hand, reduced expression of *PLC*β*4* was reported in pancreatic islets of diabetic cadaver donors compared to non-diabetic controls (12).

The observed positive correlation of *PLCXD3* with *GLP1R* is interesting (Figure 2F). Previous studies have shown that *GLP1R* receptor positively influenced β-cell function by regulating postprandial glucose level via insulin release and increased the generation of cAMP (26–28). Likewise, ablation of *PLC*ε*1* has been shown to impair GSIS through the cAMP pathway and to increase in insulin secretion via responding to incretin hormones like *GLP1/GLP1R* (24, 25). On the other hand, the inverse correlation of *PLCXD3* and BMI goes in line with a previous study reporting that a mutation in *PLCXD3* could potentially cause ROHHAD (10).

The present study shows that silencing of *PLCXD3* in INS-1 (832/13) cells reduced the insulin secretion in response to glucose as well as the insulin content without affecting cell viability or apoptosis. The findings of expression downregulation of *INS1* and *INS2* and the transcription factors that activate the insulin gene promoter (*PDX1, MAFA, NEUROD1*, and *NKX2.2*) (29–32) are further support that *PLCXD3* is an important player in insulin secretion and biosynthesis.

The finding that silencing of *PLCXD3* resulted in reduced expression of GLUT2 and GCK in INS1 (832/13) cells (Figures 4C,D) is interesting. GSIS is initiated by glucose uptake mostly by glucose transporters (GLUT2 in rodent or GLUT1 in human) (33, 34), which was then phosphorylated by GCK and subsequently metabolized through the glycolytic pathway, leading to the activation of the mitochondrial metabolism and the generation of ATP and glutamate, which trigger insulin granule exocytosis (35).

Thus, *GLUT2* and *GCK* are key players of the glucose-sensing machinery, which help pancreatic β-cells to respond to physiological blood glucose changes (36, 37). Defects in the glucose-sensing machinery have been shown to impair insulin secretion and subsequently the development of severe hyperglycemia (38, 39).

Insulin signaling in β-cells is essential to maintain normal cell function, where any defects can lead to a reduction in insulin synthesis and secretion (40). Our data showed a reduction in mRNA expression of *INSR* and protein level of *INSR*. Also, the protein expression of *AKT1*/*2*/*3* and phosphorylated *IRS2* was reduced in *PLCXD3*-silenced cells (Figure 4C). It has been reported that knockdown of *INSR* in pancreatic β-cell diminished GSIS, expression of insulin, insulin content, and expression of *PDX1* as well as *GLUT2* (40, 41). Our data go in line with previous information where inhibition of PLC activity by U73122 had caused a reduction in insulin and hormonally stimulated glucose transport along with prevention of the translocation of *GLUT4* to the plasma membrane (42). Collectively, these findings might suggest a potential role of *PLCXD3* in the development of insulin resistance by diminishing the expression of *INSR* and *GLUT2*, thus resulting in β-cell dysfunction. The microarray co-expression correlation of *PLCXD3* with the expression *GCK, GLUT2, IRS2*, and *AKT3* (Figure 4) might further support this hypothesis. However, unlike in INS-1 (832/13) cells, no co-expression correlation between *PLCXD3* and *INSR* in human islets was observed. Lack of correlation with INSR measured by microarray expression does not rule out that an internal relationship might exist. Insulin resistance syndrome including obesity, hyperglycemia, hyperlipidemia, and hypertension or insulin therapy in our donors might interfere with the insulin signaling pathway, leading to alteration of the *INSR* function (43). The main limitations of the present study are that our data do not address the expression of *PLCXD3* in sorted pancreatic β- or α-cells but rather intact human pancreatic islets. Additionally, we were not able to investigate the protein expression of *PLCXD3* in diabetic and non-diabetic human islets, which will further support the mRNA expression finding.

In conclusion, *PLCXD3* expression is among the top highly expressed PI-PLC subtypes in human islets and rat pancreatic β-cells. Reduced expression of *PLCXD3* is associated with impaired insulin secretion through a mechanism that might involve insulin receptor signaling besides altering glucose sensing.

## Data Availability Statement

All datasets generated for this study are included in the article/supplementary material.

## Ethics Statement

The studies involving human participants were reviewed and approved by the local ethics committees at both Uppsala and Lund universities (Sweden) have approved all procedures with human islets isolation and investigations. The patients/participants provided their written informed consent to participate in this study.

## Author Contributions

HA and JT designed the experiments. HA, DM, AM, SD, and NE performed all functional experimental work. HA, JT, MH, and AS analyzed human expression data. JT, HA, NS, and AS wrote and edited the manuscript.

### Conflict of Interest

The authors declare that the research was conducted in the absence of any commercial or financial relationships that could be construed as a potential conflict of interest.

## Abbreviations

T2D: type 2 diabetes
PLCXD3: phosphatidylinositol-specific phospholipase C X-domain containing 3
PI-PLC: phosphatidylinositol-specific phospholipase C
GSIS: glucose-stimulated insulin secretion
siRNA: small interfering RNA
HbA_1c_: glycated hemoglobin
BMI: body mass index
INS2: insulin 2
MAFA: MAF bZIP transcription factor A
PDX1: pancreatic and duodenal homeobox 1
INSR: insulin receptor
GCK: glucokinase
SLC2A2 (GLUT2): solute carrier family 2 member 2
HPRT1: hypoxanthine phosphoribosyltransferase 1
NEUROD1: neuronal differentiation 1
NKX2. 2: NK2 homeobox 2.
